# Helix-Loop-Helix Proteins in Adaptive Immune Development

**DOI:** 10.3389/fimmu.2022.881656

**Published:** 2022-05-12

**Authors:** Megan Aubrey, Zachary J. Warburg, Cornelis Murre

**Affiliations:** Division of Biological Sciences, Section of Molecular Biology, University of California, San Diego, San Diego, CA, United States

**Keywords:** HLH, E proteins, Id proteins, VDJ recombination, lymphopoiesis, hematopoiesis, T cell development, B cell development

## Abstract

The E/ID protein axis is instrumental for defining the developmental progression and functions of hematopoietic cells. The E proteins are dimeric transcription factors that activate gene expression programs and coordinate changes in chromatin organization. Id proteins are antagonists of E protein activity. Relative levels of E/Id proteins are modulated throughout hematopoietic development to enable the progression of hematopoietic stem cells into multiple adaptive and innate immune lineages including natural killer cells, B cells and T cells. In early progenitors, the E proteins promote commitment to the T and B cell lineages by orchestrating lineage specific programs of gene expression and regulating VDJ recombination of antigen receptor loci. In mature B cells, the E/Id protein axis functions to promote class switch recombination and somatic hypermutation. E protein activity further regulates differentiation into distinct CD4+ and CD8+ T cells subsets and instructs mature T cell immune responses. In this review, we discuss how the E/Id proteins define the adaptive immune system lineages, focusing on their role in directing developmental gene programs.

## Background

Decades of research have demonstrated the essential role of E proteins in mediating both innate and adaptive immune cell development and the wide implications of E protein activity in disease progression and immune response. In mammals, E proteins include E2A, E2-2 and HEB (1). E proteins are members of the helix-loop-helix (HLH) family of transcription factors. E proteins either homo- or heterodimerize with other HLH proteins to bind to E-box sites (CANNTG) through a basic region to modulate the expression of nearby and distal genes. This activity is opposed by Id proteins, which lack a basic DNA binding region and heterodimerize with E proteins to prevent them from binding to DNA. Together, E and Id proteins form an E-Id axis to instruct immune development.

This review discusses the role of the E-Id axis in adaptive immune development. These proteins are expressed in all mammalian cell types. They are regulated both transcriptionally and post-transcriptionally to orchestrate the development of an armamentarium of immune cell types and to establish a diverse immune repertoire. We focus on how appropriately timed differentiation to T and B cell fates is achieved while discussing how the development of alternative cell fates is suppressed.

## E-ID Axis in Early Hematopoiesis

**Figure 1:**
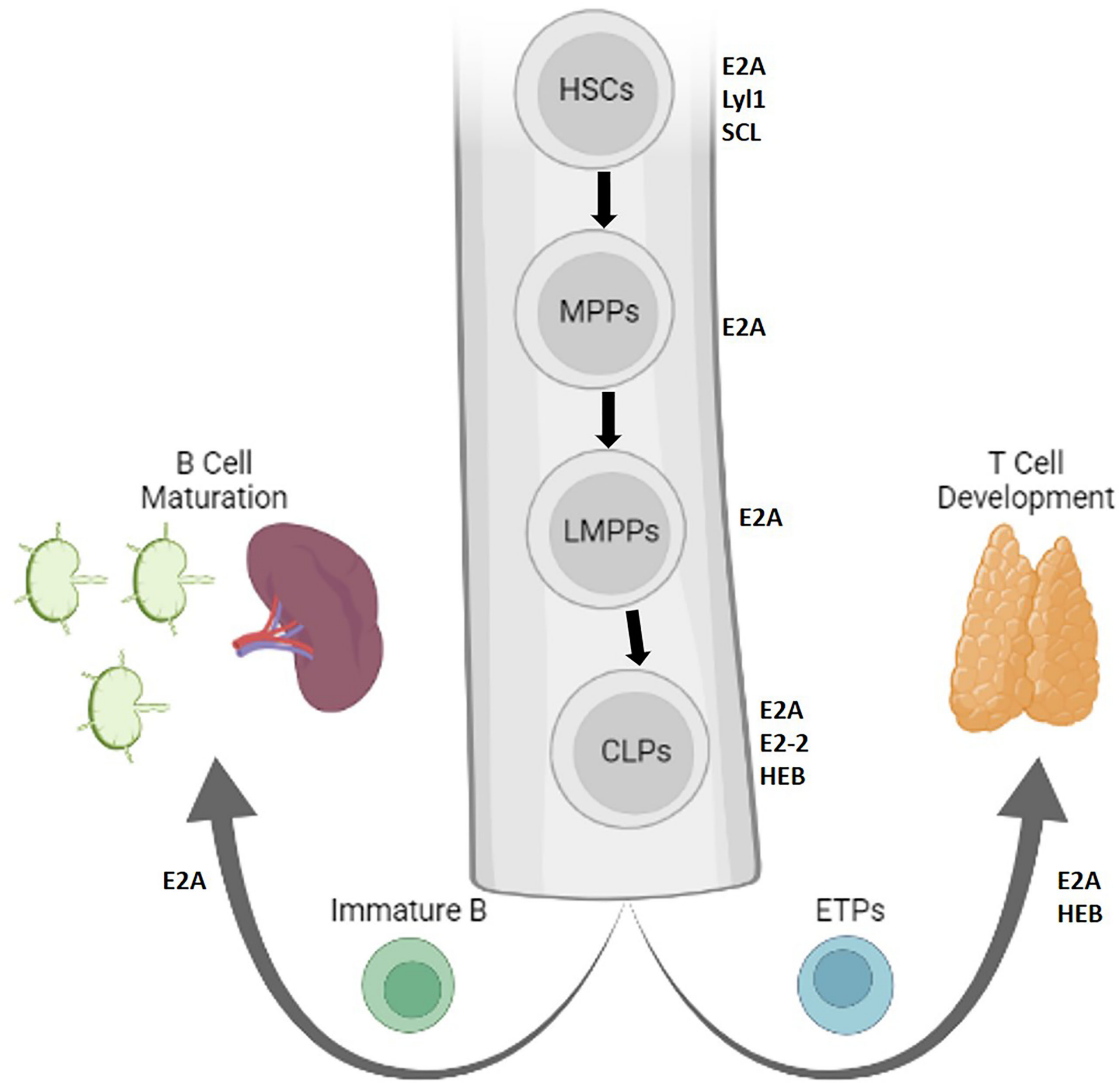
Lymphopoiesis is directed by E protein activity in early stem cells. The role of E and Id proteins in early progenitors giving rise to B and T cells is depicted in the bone marrow. Protein factors that support stem cell maintenance or self-renewal are indicated adjacent to each cell, and bolded arrows represent lineage differentiation (Created with BioRender.com).

Adaptive immune development begins in the fetal liver and in the bone marrow in adults, where E and ID proteins influence developmental decisions in hemopoietic stem cells (HSCs), which give rise to all blood cells (**Figure 1**). Differentiation to HSCs is achieved by the E protein SCL/TAL1, and maintained by E2A proteins and their repressive heterodimerizing HLH partners Lyl1 and Id1 (2–5). These factors also set the stage for the ratio of progenitors giving rise to B and T cells. E proteins oppose proliferation of HSCs, priming their expression to promote lymphoid-associated gene expression (6, 7). This activity promotes their differentiation into multipotent progenitors (MPPs) and further into lymphoid-primed MPPs (LMPPs), while preventing granulocyte-monocyte progenitor (GMP) development and partially restricting megakaryocyte-erythrocyte progenitor (MEP) development (6). As a result, E2A-deficient mice are associated with reduced HSCs and MPPs (3). Other E proteins, E2-2 and HEB, were found to be expendable at this early stage of development (8–10). Thus, E protein activity orchestrates a supportive transcriptional landscape for lymphocyte development in HSCs.

Id protein inhibition of E protein activity might play a role in generating a diverse immune repertoire from HSCs. E2A promotes HSC differentiation and represses proliferation by controlling the expression of p21 and Notch1 (11–14). E proteins further drive differentiation to LMPPs to common lymphoid progenitors (CLPs), which give rise to several cell fates including B cells, T cells, dendritic cells, innate lymphoid cells, and natural killer (NK) cells (10, 15). In the absence of E2A expression, fewer LMPPs progress to the CLP stage (6). The cells that do progress preferentially feed alternative lineages seeded by GMPs, MEPs, and Pre-MegE-progenitors (7). In the absence of E2A and HEB, CLPs are compromised in their ability to express an early lymphoid program (10). The capacity of CLPs to differentiate into NK, B or T lineages may be further divided by their expression of different surface markers (16–19). A recent study suggested that heterogeneous levels of E and Id proteins in CLPs may contribute to these unique differentiative capabilities (19). Thus, fine tuning of E protein activity in CLPs instructs immune cell fate.

## Early B Cell Development

**Figure 2:**
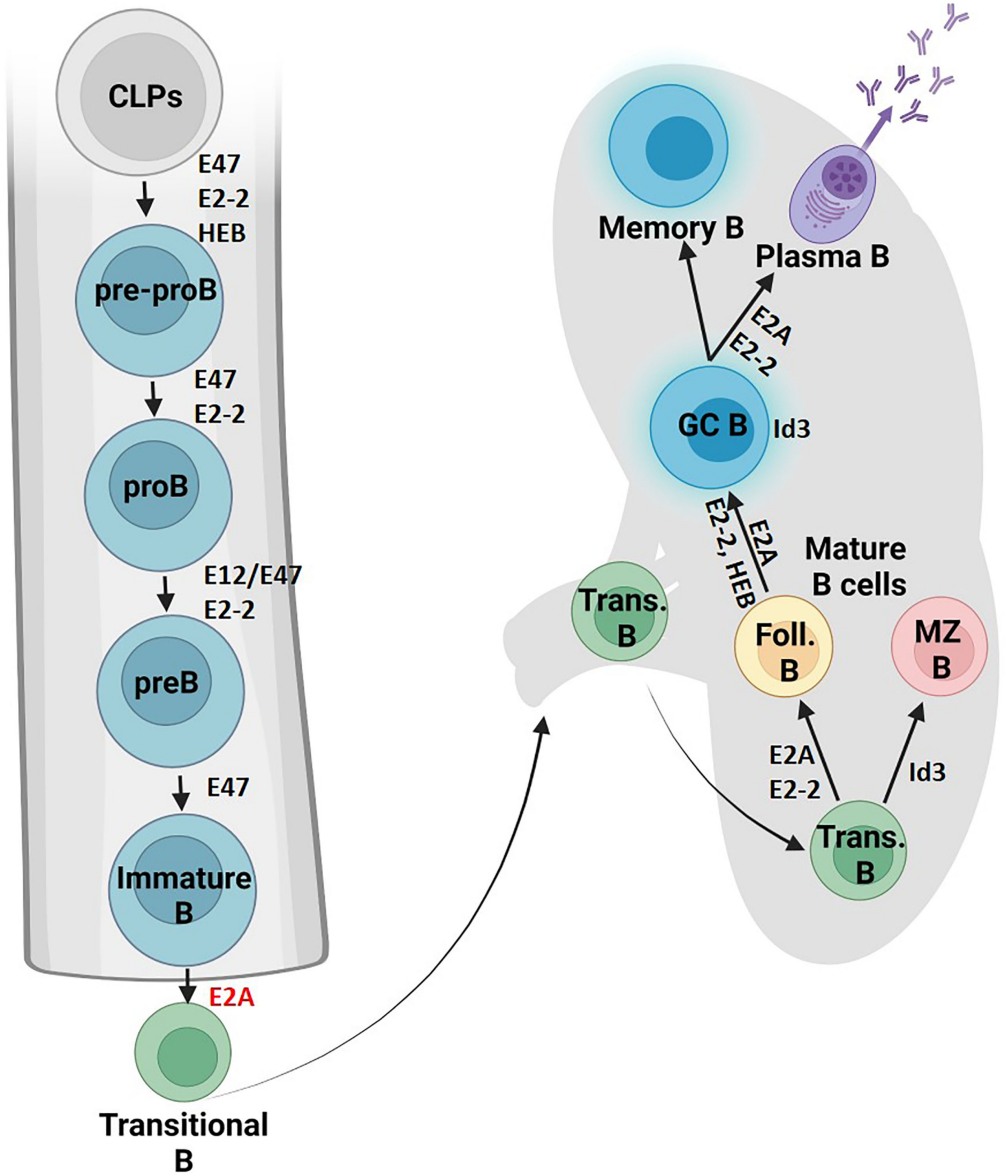
B cell fate and differentiation is directed by E protein activity. The role of E proteins and their antagonists in B cell development is shown in the bone marrow and lymphoid organs. Protein factors supportive of differentiation or cell maintenance are indicated in black font next to the arrows or adjacent to the cell respectively. Protein factors with repressive functions are indicated in red font (Created with BioRender.com).

E proteins orchestrate B cell development by defining signaling pathways in CLPs (**Figure 2**). E2A activity is required to activate Ebf1 and IL7 receptor protein (IL7Rα) in CLPs, which together with E2A activate Pax5 (20–23). E2A proteins also act in concert with Ebf1 to induce Foxo1 expression (24). Subsequently, E2A and HEB coordinate with Foxo1, Pax5 and Ebf1 to support the progression of CLPs through the B cell lineage (23, 25). Aberrant Id3 expression at this earlier stage alternatively arrests B cell development and prevents IL7Rα induction, later inducing caspase-mediated apoptosis (26). Cytokine signaling from the TGF-β family represents one mechanism by which Id3 expression is regulated (27). Thus, E proteins promote B cell development from CLPs by priming a B-cell transcriptional network.

E proteins then stimulate the subset of CLPs primed with B cell lineage genes to progress into pre-pro-B cells. Studies have implicated that the E2A isoform E47 is required to get past this stage, while E12 is dispensable (28–31). E2-2 and HEB also both contribute to early B cell development (32). Notably, E2-2 mRNA expression is particularly high in pro-B cells and orchestrates the developmental maturation of these early B cell progenitors into pre-B cells (33).

In the B cells, E proteins are regulated by lineage specific post-transcriptional mechanisms. E47 homodimers, for example, are only detected at high levels in B cells (34). This cell specific homo-dimer activity may involve phosphorylation of specific residues of the E2A proteins (34, 35). Histone acetyltransferases including p300, CBP, and PCAF interact with E2A to mark the epigenetic chromatin landscape (36, 37). Further, miRNAs regulate E protein activity. A recent study identified miR-191 as a rheostatic regulator of B cell development to modulate E2A mRNA abundance from pro-B to immature B cells (38).

E proteins regulate gene expression by coordinating changes in nuclear architecture. E2A occupancy at the Ebf1 locus is associated with relocation away from the nuclear lamina in pro-B cells (39). E2A binding at or near enhancers or promoters was associated with deposition of active chromatin markers such as H3K4me1 with further activating epigenetic alterations between pre-pro-B to pro-B cells (40). Activated genes with E2A occupancy frequently contained coordinated DNA binding with Ebf1, Foxo1 and CTCF. Recent studies indicated that E2A occupancy is closely associated with recruitment of members of the cohesin complex (41). In parallel studies it was revealed that H3K27Ac marked enhancers are closely associated with recruitment of cohesin (42–44).

Collectively, these studies suggest that E2A may act, at least in part, by initiating loop extrusion across the enhancer landscape. Other mechanisms may also act with E2A to promote B cell development. Notably, E2A recruits Tet2 and Tet3 to promote chromatin accessibility adjacent to E-box binding sites in pro-B cells (45). Future studies are warranted to determine how E2A mediated changes in DNA methylation and recruitment of cohesin are linked to induce lineage specific gene programs.

E proteins may also be essential in preventing the premature progression of B cells from pre-pro-B into pro-B cells. E2A enforces this checkpoint by binding an E-box element in a p21 regulatory region, which encodes a CDK inhibitor and induces cell cycle arrest (11). This checkpoint can be circumvented by repression of E protein activity by Id1-3 (11, 26). The downregulation of E protein activity by rapid induction of Id proteins occurs upon successful heavy chain V-DJ rearrangement in pro-B cells, and it will be important to establish whether and how alterations in Id3 protein levels modulate E2A activity to orchestrate antigen receptor assembly (28).

## Generation of B Cell Diversity

Somatic recombination events in the B cell lineage generate a diverse antibody repertoire. B cells rearrange the variable regions of their immunoglobulin heavy chain (Igh) and immunoglobulin light chain (Igk or IgL) loci to produce mature B cells with unique antigen binding specificities. E2A regulates these recombination events by controlling appropriate expression of the Rag genes, as well as chromatin accessibility and 3D spatial organization of the Igh and Igk loci. Following these recombination events, B cells can undergo class switch recombination (CSR) and somatic hypermutation (SHM) to generate antigen receptors with higher affinities for their cognate antigens. E proteins regulate CSR and SHM by promoting chromatin accessibility at the targeted immunoglobulin (Ig) genes and by controlling the expression of key enzymes involved in these processes.

### E2A Regulates Rag Expression in the B Cell Lineage

Recombination is catalyzed by the recombinase activating genes, Rag1 and Rag2 (46, 47). Rag1/2 expression peaks twice in the B cell linage, first in pro-B cells during Igh rearrangement, when E47 expression is high. Rag expression is then downregulated as cells pass through the pre-BCR checkpoint and transition to pre-B cells (48). E protein activity declines during this time, as pre-BCR signaling upregulates Id3 expression and E47 protein levels decline (49, 50). Rag and E47 protein levels are elevated again in pre-B cells undergoing Ig light chain rearrangements (48, 49). In E2A deficient mice, B cell development is blocked at the pre-pro-B cell stage and Igh rearrangements fail to initiate due to lack of Rag activity (31, 51). E2A regulates Rag expression in a dose dependent manner (49).

The Rag1/2 genes share a single genetic locus. An evolutionarily conserved B cell specific enhancer of Rag (*Erag*) contains E-box binding sites. Deletion of *Erag* in mice reduces Rag1/2 expression and compromises D_h_-J_h_ and V_h_-D_h_J_h_ recombination (52). Recent findings indicate that E2A directly regulates Rag1/2 gene expression in pro-B cells by binding to the Rag promoters and enhancer and orchestrating chromatin conformations that promote a transcriptionally active Rag locus (41). E2A also binds two additional B cell specific regulatory elements (R1B and R2B), which partially overlap with *Erag*. R1B and R2B orchestrate a B cell specific chromatin architecture at the Rag1/2 gene cluster. Deletion of these E2A binding elements resulted in reduced chromatin accessibility of the Rag1/2 genes, a loss of genomic interactions across the locus, reduced Rag1/2 expression, a significant developmental block at the pro-B cell stage, and severely compromised Igh recombination. Further, E2A directly regulates the Rag1 promoter in pro-B cells. Specific mutation of all 7 E-box sites in the Rag1 promoter (R1pro-E-box^mut/mut^), resulted in a loss of chromatin accessibility at the Rag1 gene and reduced Rag1 gene expression. R1pro-E-box^mut/mut^ mice are phenotypically similar to Rag^-/-^ mice, have severely compromised Igh V-D_h_J_h_ recombination, and exhibit a developmental block at the pro-B cell stage (41).

### Recombination of the Igh Locus

The immunoglobulin heavy chain locus is comprised of V_h_, D_h_, and J_h_ genes, which recombine in a step-wise manner. D_h_ to J_h_ recombination occurs first, and is followed by V_h_ to D_h_J_h_ recombination (53). E2A regulates V_h_(D_h_)J_h_ recombination by promoting chromatin accessibility at the Igh locus. Early studies found that ectopic expression of either E2A gene product (E12 or E47) along with Rag1/2 in non-B lineage cells is sufficient to initiate Igh germline transcription (GLT) and D_h_ to J_h_ recombination (but not V_h_ to D_h_J_h_ recombination) (30, 54, 55). E2A initiates and maintains Pax5 expression in pro-B cells and cooperates with Pax5 to promote further chromatin accessibility and allow V_h_ to D_h_J_h_ recombination (29). Ectopic expression of Pax5 with Rag1/2 and E2A in non-lymphoid cells is sufficient to induce V_h_ to D_h_J_h_ recombination (56). Interestingly, enforced Pax5 expression restores Rag expression and D_h_-J_h_ recombination at the heavy chain locus in Vav-CRE E2A^fl/fl^ mice, even though the Rag1 promoter is directly regulated by E2A (29, 41).

E2A is also essential for Igh locus contraction (57, 58). Prior to V_h_(D_h_)J_h_ recombination, the Igh locus repositions from the lamina to the nuclear interior and undergoes contraction to bring V_h_ and D_h_J_h_ genes into close physical proximity (59). This compaction allows V_h_ and D_h_J_h_ genes to adopt a wider spectrum of configurations in pro-B cells, to promote a higher diversity of V_h_ genes in the antibody repertoire (58). E2A directly binds to PAIR elements, regulatory elements in V_h_ region that facilitate locus contraction (60–63). The role of E2A binding at PAIR elements is not yet clear but it likely involves recruitment of the cohesin machinery to initiate loop extrusion across the Igh locus.

Expression of a pre-B cell receptor (pre-BCR) composed of a rearranged Igh protein and a surrogate light chain (SCL) is a developmental checkpoint that monitors for successful rearrangement of an Igh allele. Pre-BCR signaling indicating a productive Igh chain has recombined enforces allelic exclusion of the Igh locus. Pre-BCR mediated regulation of E2A might be important for downregulation of the SLC genes, as well as other pre-BCR co-receptors and downstream signaling proteins (64, 65).

### Recombination of the Igk Locus

The roles of E2A in orchestrating recombination of the immunoglobulin kappa (Igk) locus have been extensively studied. The E2A proteins were initially identified in a screen for factors that bind sites across the kappa locus intronic enhancer (iEκ) (66). Analogous to its role in Igh recombination, E2A promotes chromatin accessibility at the Igk locus. Ectopic E2A expression, along with Rag1/2, is sufficient to induce Igk germline transcription and V_k_ to J_k_ rearrangements in non-lymphoid cells (55). While forced Pax5 expression restored D_h_-J_h_ recombination at the Igh locus in E2A^-/-^ mice, it did not rescue V_k_-J_k_ rearrangements, indicating a unique role for E2A in promoting Igk locus assembly (29).

E2A proteins directly bind sites across the Igk locus to recruit the histone acetyltransferases CBP and p300 (67, 68). E2A may increase the rearrangement frequencies of V_k_ genes by promoting their transcription. Promoters bound by E2A or that contain E2A binding sites are associated with strong promoters and are expressed at significantly higher frequencies compared to overall V_k_ genes (39, 67, 69). Another possible mechanism may involve recruitment of cohesin to instruct loop extrusion at enhancers across the Igh locus akin to that described above for the Igh locus. The Igk locus contains an ensemble of enhancers including, the intronic enhancer (iEκ) and the 3’ enhancer (κE3’), that regulate V_k_-J_k_ rearrangement (70–72). E2A binding to iEκ is essential for enhancer activation and regulates the appropriate developmental timing of Igk recombination (73–75). Mutation of two of the three E-box sites in iEκ resulted in the same reduction in Igk rearrangement as that with deletion of the entire iEκ enhancer (73). Before initiating light chain rearrangements, large cycling pre-B cells attenuate their IL-7/STAT5 signaling, cell cycle exit and transition into resting small pre-B cells. IL-7/STAT5 signaling negatively regulates Igk recombination by antagonizing E2A binding at iEκ (74, 75). Similar mechanisms regulate the activation of kE3’. Developmental control of the kE3’enhancer involves both active stimulation by PU.1, IRF4, and E2A and repression by STAT5. STAT5 signaling reduces kE3’ activity in pro-B cells, possibly by blocking PU.1 recruitment to the enhancer, as STAT5 and PU.1 competitively bind to the enhancer (76). Pre-BCR signaling induced IRF4 promotes kE3’ activation by cooperatively binding to the enhancer with E2A and by rendering kE3’ activity insensitive to STAT5 (76–79). E2A and PU.1 recruit the TET proteins to kE3’ where they promote increased chromatin accessibility by facilitating DNA demethylation (45). Proper developmental timing of Igk locus demethylation appears critical for appropriate Igk recombination. Proximal V_k_ gene promoters and kE3’ were hypomethylated in mice in which the *de novo* methyltransferases Dnmt3a and Dnmt3b were deleted. These mice undergo premature Igk rearrangements, have increased Igk rearrangement frequencies, and over-utilize their most proximal V_k_ genes (80).

**Figure 3:**
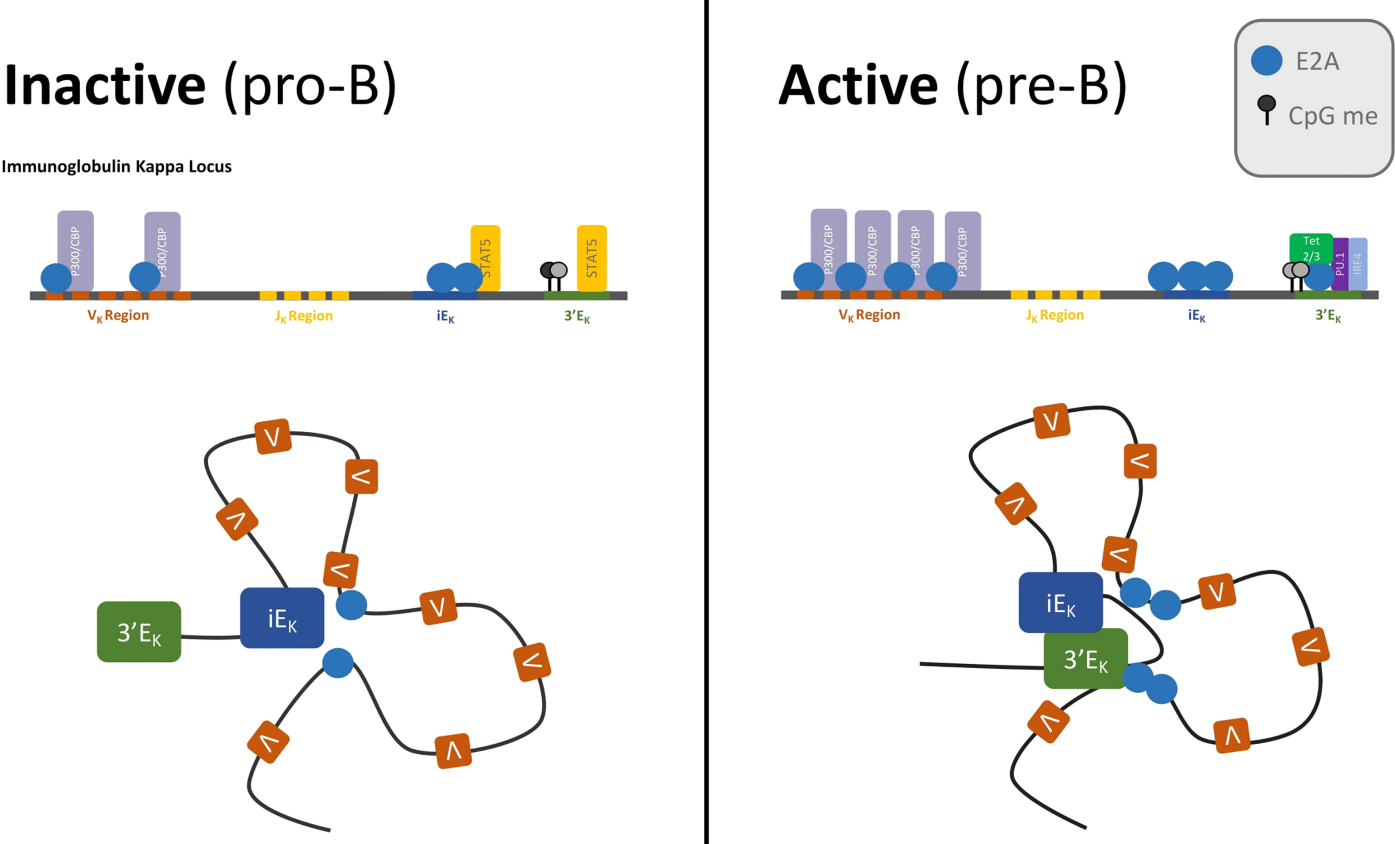
Enhancer activation by E2A drives IgK recombination.The IgK locus is poised for recombination in pro-B cells—when E2A is bound to V_K_ genes, V_K_ regions have acetylated histone marks, and the locus is already contracted, with iE_K_ making extensive contacts with the V_K_ region. Activation of the locus coincides with E2A activation of the IgK enhancers. Pre-BCR signaling and IL-7/STAT5 attenuation render the iE_K_ and 3’E_K_ enhancers insensitive to STAT5. E2A occupancy at the enhancers increases, and the Tet proteins are recruited to 3’E_K_ where they demethylate CpG residues. The now accessible 3’E_K_ enhancer forms extensive contacts with the V_K_ region.

The Igk locus is poised for V_k_J_k_ recombination in pro-B cells, where it already exhibits signs of chromatin accessibility and has already undergone large scale locus contraction. The Igk locus repositions to the permissive compartment and contracts at the pre-pro-B to pro-B developmental cell transition. During this transition, the intronic enhancer (iE_k_) forms extensive contacts with V_k_ genes across the locus that are associated with E2A occupancy. These changes in chromatin conformation are accompanied by increased Igk transcription, widespread H3K4 demethylation, and E2A binding across the locus (39, 81, 82). In response to pre-BCR signaling, the locus further contracts. E2A occupancy at the locus increases and kE3’ forms stronger chromatin interactions with the V_k_ region (**Figure 3**). Interactions between kE3’ and Igk flanking regions are reduced, while interactions between kE3’ and V_K_ genes that are located close to E2A binding sites increase. There are strong positive correlations between presence of E2A binding sites, V_k_ gene usage, and long-range chromatin interaction frequencies between V_k_ genes and the kappa regulatory elements, which suggest that E2A is a key factor in Igk locus contraction (82).

In conclusion, much has been learned about the roles of E2A in orchestrating Igk locus rearrangement. In our view the most appealing mechanism is that the E2A proteins bind enhancer elements across the Igk locus to deposit H3K27Ac across the enhancer repertoire. The deposition of H3K27Ac may then act to sequester chromatin remodelers like Brg1 that in turn sequester cohesin to initiate loop extrusion (42). Thus, a common theme is now emerging in which transcription factors, like E2A, sequester cohesin to promote large-scale alterations in chromatin folding, enabling V_k_ regions to encounter J_k_ elements with distinct frequencies that are independent of genomic separation.

## Mature B Cell Development

After successful VDJ rearrangement and receptor editing of the Ig light chain genes, E and Id proteins further instruct the development of pre-B cells. This transition is mediated by upregulation of Id3 and a reduction in E protein abundance triggered by BCR signaling (49). E47 levels therefore decline in transitional B cells followed by a near complete loss of E47 expression in mature B cells. Genetic studies showed that high levels of E2A promote follicular B cell development while high Id3 abundance favors the marginal zone B cell fate (33, 49). E2-2 serves an overlapping role controlling this developmental decision as revealed by the transfer of E2A- and E2-2-deficient fetal liver cells into irradiated Rag-deficient mice (33). E protein activity is also essential for the development of germinal center and plasma cells (83). Likewise, in the absence of Id3 expression germinal center B cell development is severely affected (84). Specifically, when researchers abrogated Id3 expression in germinal center B cells, the expression of genes encoding for components of antigen receptors, cytokine receptors, and chemokine receptors was severely perturbed (83). E2A and E2-2 activity is also essential for the developmental progression of plasma cells (29, 84, 85). E2A and E2-2 promote plasma cell identity by directly activating Blimp1 and Xbp1 expression (84, 85). Together these studies show that HLH proteins play instrumental roles in orchestrating the response of B cells to exposure of infectious agents.

### Class Switch Recombination and Somatic Hypermutation

Activation of mature naïve B cells initiates class switch recombination (CSR) and somatic hypermutation (SHM). E proteins regulate both CSR and SHM by 1) transcriptionally regulating key factors involved in these processes, 2) interacting directly with CSR and SHM proteins and targeting them to Ig genes, and 3) by increasing the chromatin accessibility of Ig genes.

The enzyme activation induced cytidine deaminase (AID) is required for both CSR and SHM. AID deaminates cytosine bases to uracils. In CSR, the DNA repair factor UNG then excises these uracil bases and DNA repair factors convert these SSBs to DSBs (86). In SHM, mutations are generated by a variety of error prone DNA repair mechanisms that are employed to repair the mismatched U:G bases (87). E2A and E2-2 directly promote expression of AID by binding to regulatory elements in the *Aicda* locus (the AID gene) and increasing chromatin accessibility of enhancer elements (85, 88, 89). Loss of E protein activity in activated B cells inhibits CSR, due in part to loss of AID expression (85, 90, 91). CSR to IgG1 expression is blocked in in E2A/E2-2 DKO mice due to loss of AID expression (85). Overexpression of Id2 reduces AID expression in activated B cells (92). However, a balance of E protein activity must be maintained for normal CSR, as Id2 also plays an inhibitory role in CSR. Id2 deficient B cells undergo CSR to IgE at a much high frequency than that of wild-type B cells (93).

E2A proteins also bind directly to Ig genes to promote SHM and CSR. E2A forms a complex with AID, Pax5, ETS1 and IRF4 that functions to target AID to sites within the Igh locus (94, 95). E2A and E2-2 promote CSR by opening chromatin at the 3’RR enhancer and activating GLT of switch regions. E2A/E2-2 DKO mice have impaired CSR to IgE, due to loss of activation of the 3’RR enhancer and IgE GLT (85). Further, E-box binding sites within Ig enhancers promote efficient SHM (96–99). E2A may help direct AID to DNA, and genome wide E2A occupancy is associated with AID targeting (99). Finally, we suggest that E2A proteins act to promote CSR and SHM by initiating loop extrusion across the switch regions and V gene segments and note that E2A likely plays an additional role in promoting phase separated droplets to orchestrate CSR and SHM.

## Regulation of Early T Cell Development by HLH Proteins

**Figure 4:**
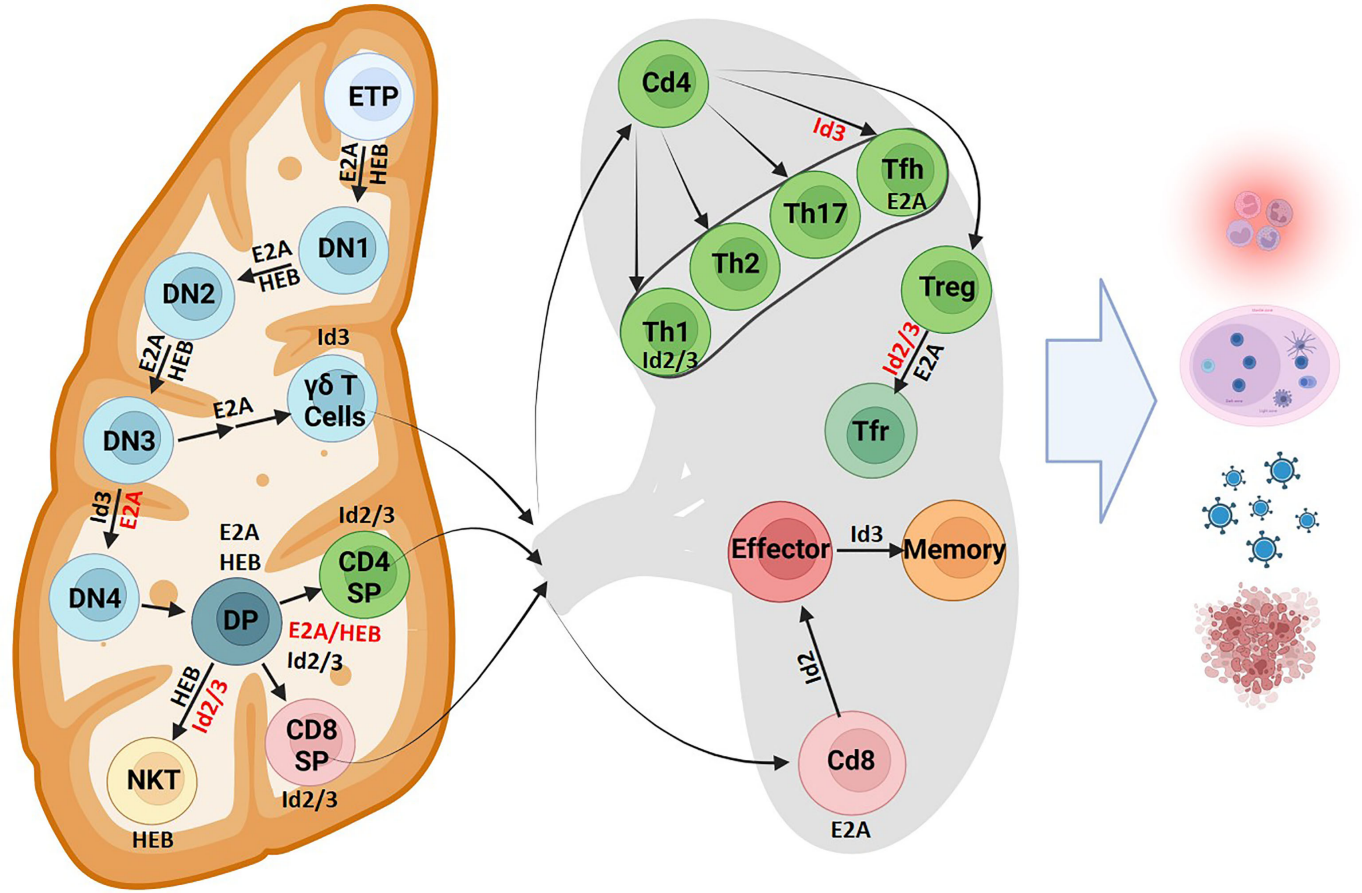
T cell fate and differentiation is directed by E protein activity. The role of E proteins and their antagonists in T cell development is shown in the thymus and lymphoid organs. Protein factors supportive of differentiation or cell maintenance are indicated in black font next to the arrows or adjacent to the cell respectively. Protein factors with repressive functions are indicated in red font (Created with BioRender.com).

A fraction of LMPPs develops into early T progenitors (ETPs) that then home to the thymus (**Figure 4**). E2A and HEB promote homing by modulating chemokine receptor expression, including CXCR4, to direct thymocytes to the cortex (100). Here, ETPs encounter Delta-class Notch ligands. An ensemble of genes involved in Notch signaling are directly activated by E47 (21). Together with E2A, Notch signaling prevents the activation of B-lineage and myeloid factors and promotes T lineage development. These functions are opposed by Id1 and Id2 expression in these early progenitors and in double negative (DN) T cells, promoting an innate lymphoid fate instead. T cell progression was blocked in CLPs with disrupted E2A and HEB activity, instead favoring differentiation to alternative lineages (101). Once ETPs migrate to the thymus, E2A and HEB, in coordination with Notch signaling instruct further development (21, 32, 102–104). In developing thymocytes, E proteins modulate the expression of gene programs including those involved in cell cycle progression, pre-TCR signaling and cytokine gene expression (105). Prominent amongst the genes activated by E47 expression are CDK6, Socs1/2, Ets, Foxo1, and GATA3. The E2A proteins may act coordinately with Bcl11b, another key factor known to establish T cell identity, to activate a common set of target genes (106, 107). Researchers have found that E2A binds regulatory elements in a distal long non-coding RNA, ThymoD (108). ThymoD transcription is initiated from within the Bcl11b intergenic region where it acts to promote T cell commitment by repositioning the Bcl11b enhancer from a heterochromatic environment at the lamina to the euchromatic compartment located in the nuclear interior (107, 108). The overlapping gene expression profiles between E2A, Bcl11b, and ThymoD knockout mice combined with the evidence of E2A binding to elements within ThymoD implicates the possibility that E2A could indirectly initiate Bcl11b expression by modulating non-coding transcription. A prominent Bcl11b target in developing thymocytes is Id2. Interestingly, the majority of genes regulated by Bcl11b are also modulated by Id2 (104, 109). HEB also performs multiple, unique functions in thymocyte development. Elegant studies revealed that an alternatively spliced form of HEB, named HEBAlt, increases the development of T cell progenitors (110, 111). Subsequent studies showed that in the absence of HEB T cell progenitors adopt alternative cell fates (112). HEB also directly activates the expression of pre-Tα, a component of the pre-TCR complex (113). Thus, a detailed picture is now emerging in which E2A and HEB act collaboratively to orchestrate the development of early T cell progenitors.

## Generation of T Cell Diversity

T lymphocytes rearrange their TCRα, TCRβ, TCRγ and TCRδ loci to generate diverse T cell receptor repertoires. T cell receptor rearrangements initiate at the CD4^-^ CD8^-^ double negative (DN) stage of thymocyte development. TCRβ, TCRγ and TCRδ rearrangements all occur simultaneously in DN2 cells. Successful rearrangement of TCRγ and TCRδ loci results in expression of a γδ TCR and potential development into the γδ T cell lineage. Successful rearrangement of a TCRβ chain results in expression of a pre-TCR. Pre-TCR signaling allows progression to the DN4 cell stage where TCRα rearrangements occur, so that cells may become αβ T cells. The E-ID axis plays critical roles in T cell development by regulating rearrangement events at all four TCR loci, orchestrating the αβ versus γδ cell fate decision, and by enforcing key developmental checkpoints.

### αβ/γδ T Cell Lineage Decisions

The E-ID axis determines whether cells adopt the αβ or γδ T cell fate. γδ TCR signaling strength is a critical determinant in the choice to become αβ or γδ T cells. Stronger γδTCR signals favor lineage commitment to the γδ T cell fate, while weaker γδ TCR signals favor commitment to the αβ T cell fate (114, 115). γδ TCR signals mediate lineage decisions through activation of the Erk-Egr-Id3 pathway (114, 116). In response to TCR signaling, Egr induces a level of Id3 expression that is proportional to the TCR signaling strength, and Id3 expression levels correlate with commitment to the γδ cell fate. Cells committed to the γδ cell fate display higher levels of Id3 expression (114). Id3 plays a critical role in γδ/αβ lineage fate decision. Loss of Id3 decreases the number of γδTCR+ cells, however Id3 overexpression does not increase the number of γδTCR+ cells or influence their maturation (114). Thus, Id3 is necessary but not sufficient to drive the γδ cell fate. Egr acts upstream of Id3 to promote the γδ cell fate, but likely also effects other pathways besides Id3 to promote γδ T cell development, as Egr1 overexpression is sufficient to increase the frequency of γδ T cell (114, 116). The increase in γδ T cells depends in part, on Id3 activation, as the Egr1 overexpression phenotype is diminished in an Id3 deficient background (116). Id3 regulates the γδ/αβ lineage fate decision by promoting the survival of γδ T cells and repressing the survival of αβ T cells in response to strong TCR signals. In response to a strong γδ TCR signal, Id3 deficiency increases the expression of the anti-apoptotic protein Bcl-XL and the survival of cells committed to the αβ lineage, while reducing expression of the anti-apoptotic protein Bcl-2 in mature γδ T cells (116).

### The E-ID Axis Regulates Rag Expression in the T Cell Lineage

There are two waves of Rag expression in developing T cells. The first wave peaks in DN cells at a time when the TCRβ, TCRδ, and TCRγ loci rearrange. The second wave peaks in double positive (DP) T cells during the course of TCRα rearrangements (117). The E-Id protein axis is critical to coordinate these waves of Rag expression. The E proteins, E2A and HEB, positively regulate the expression of Rag1 and Rag2 in DN and DP cells (30, 37, 118). Many studies have sought to characterize the cis-regulatory elements that regulate Rag expression in thymocytes (41, 119, 120). Rag1 and Rag2 share a single genetic locus and their expression in T cells depends on two overlapping cis regulatory elements, the Rag-T cell enhancer (*R-TEn*) and the anti-silencer element (ASE). E2A directly binds to E-boxes in *R-TEn*, which is located within ASE, as well as the Rag1 promoter and upregulates Rag1/2 expression by coordinating or maintaining the assembly of a transcriptionally active chromatin hub at the Rag locus in both DN and DP cells. Deletion of *R-TEn* induces developmental blocks at the DN3 and DP cell stages (41, 120). Following productive TCR rearrangements, Id3 protein expression is upregulated in response to pre-TCR signaling, positive selection and γδ TCR signaling (116, 121, 122). Enforced expression of Id3 in T cell progenitors reduces levels of Rag1 and Rag2 (123). These studies suggest that Id proteins function to promote allelic exclusion by antagonizing E2A binding at the Rag locus, which downregulates Rag1/2 expression and thus prevents further TCR rearrangements (124).

### TCRβ Rearrangement

In addition to positively regulating Rag expression, high E protein activity in early thymocyte development promotes TCRβ rearrangements by increasing chromatin accessibility of the locus. The murine TCRβ locus is composed of V_β,_ D_β_, and J_β_ genes. The locus recombines in a step-wise manner, with D_β_ to J_β_ rearrangement occurring before V_β_ to D_β_ J_β_ rearrangement (125). Recombination of the TCRβ locus is dependent on the TCRβ enhancer (Eβ), which drives germline transcription at and promotes chromatin accessibility of the D_β_-J_β_ gene clusters (126–129). In DN thymocytes, E2A binds to conserved E-box binding sites in Eβ, the Dβ2 promoters, and the majority of Vβ promoters and drives germline transcription from Vβ promoters as well as H3 histone acetylation at Vβ, Dβ and Jβ genes in dosage dependent manners, likely by directly binding to and recruiting the histone acetyl transferases CBP and p300 (37, 68, 130, 131).

E2A deficient and null mice have reduced numbers of thymocytes, exhibit a partial block in thymocyte development at the DN1 stage, and display gene dosage dependent deficiencies in both D_β_-J_β_ and V_β_-D_β_J_β_ rearrangements (37, 103, 132). HEB plays a modest role in TCRβ recombination. HEB deficient mice show dosage independent deficiencies in V_β_ germline transcription, and do not display a partial developmental block until the ISP stage (113, 131). It is possible that the modest defects in rearrangement seen in E2A and HEB deficient mice are caused by loss of a single E protein being compensated for by homodimers of the remaining E-protein (133, 134). Studies designed to address concerns of compensation generated mice with double conditional knockouts of HEB and E2A at an early stage in lymphocyte development (HEB^fl/fl^ E2A^fl/fl^ Lck^+/Cre^), as well as mice that express a dominant negative HEB gene (HEB^bm/bm^), which contains a mutation in the DNA binding region of HEB and forms non-functional heterodimers with E2A (134, 135). Studies with these mice confirmed that E2A and HEB can partially functionally compensate for one another. Both HEB^fl/fl^ E2A^fl/fl^ Lck^+/Cre^ and HEB^bm/bm^ exhibit severe developmental blocks at the DN stage, and HEB^bm/bm^ show severely impaired V_β_-D_β_J_β_ rearrangements (134, 135). Together, these data show that the E-proteins play essential and overlapping roles in controlling TCRβ locus assembly.

### β-Selection

Successful rearrangement of a TCRβ chain results in expression of a pre-TCR containing the TCRβ chain and a surrogate light chain TCRα (pre-Tα). Pre-TCR signaling indicates rearrangement of a productive TCRβ allele, ensures allelic exclusion by blocking further TCRβ rearrangement, and allows cells to transition past the β-selection checkpoint and develop into DN4 and DP cells. The E-Id axis acts on many levels to regulate proper development at the β-selection checkpoint. E47 and HEB regulate pre-Tα expression in a dose sensitive manner (135, 136). E2A and HEB double conditional knockout mice exhibit a severe developmental block at the DN3 stage, exhibit normal TCRβ rearrangements, but have reduced pre-Tα expression, suggesting that this block could be due to lack of pre-Tα protein (135).

The E-Id axis also regulates the proliferation of thymocytes before and after β-selection. Prior to β-selection, E2A activity suppresses IL-7 induced proliferation. DN3 cells engaging in TCRβ rearrangement are cell cycle arrested in G1. After productive TCRβ rearrangement, pre-TCR signaling induces many rounds of proliferation as cells transition to DN4 and DP stages. HEB and E2A are necessary to keep DN3 cells in a low or non-proliferating state prior to pre-TCR signaling (135, 137, 138). Pre-TCR signaling inhibits E protein activity primarily by inducing expression of Id3 and by promoting E2A degradation. This loss of E protein activity then allows proliferative expansion of DP thymocytes. Upregulation of Id3 and silencing of E protein activity functions to ensure allelic exclusion by reducing E2A occupancy at the TCRβ enhancer and Vβ regions, as well as CBP and H3 acetylation at Vβ regions. Inhibition of E2A is essential for allelic exclusion. Enforced expression of E47 in DN thymocytes that already contain a functional TCRβ transgene enables continued rearrangement of the TCRβ loci (37). Further, E2A is essential to enforce the β-selection checkpoint. E2A deficiency allows thymocytes that have not undergone TCRβ rearrangement to bypass selection and develop into DP and even single positive thymocytes (137, 138).

### TCRα Rearrangement

The TCRα and TCRδ genes share a single genetic locus, with the TCRδ genes nested within the TCRα genes, such that rearrangement of the TCRα gene results in deletion of the entire TCRδ gene (139). Rearrangements of the TCRα/δ locus are regulated by two enhancers, E_α_ and E_δ_ (140). In DP cells, pre-TCR signaling deactivates the E_δ_ enhancer, activates the E_α_ enhancer, and promotes the formation of a chromatin hub in which CTCF and cohesin mediate long range chromatin interactions between E*α*, the more proximal 3’ Vα/δ and the more 5’ distal Jα promoters and drives germline transcription (141). The E*α* enhancer contains three E-boxes, two of which are occupied by E2A prior to pre-TCR signaling. The third E-box site is bound by E2A only in DP cells and is not occupied in HEB^-/-^ cells, which suggests that this site is bound by a E2A-HEB heterodimer (142). E*α* does not drive TCR*α* expression in mature αβ T cells and is inactivated following positive selection. Following E*α* inactivation the TCR*α* chromatin hub dissolves. There is a loss of long-range enhancer-promoter interactions, activating histone modifications (H4K3me1 and H4K3me3), and E2A and HEB binding to the enhancer (143).

### TCRγ Rearrangement

E2A and HEB promote TCRγ rearrangements. E2A and HEB are each sufficient to initiate TCRγ rearrangements in non-lymphoid cells expressing Rag1 and Rag2 (144). The TCRγ locus is composed of 3 functional clusters: Cγ1, Cγ2, and Cγ3. Rearrangement of the Cγ1 cluster has been the most extensively studied. The Cγ1 cluster contains four Vγ genes and one Jγ gene (Jγ1). Vγ genes in the Cγ1 cluster rearrange with Jγ1 in a developmentally ordered manner. The more proximal Vγ3 and Vγ4 rearrange in early fetal thymocytes, while the more distal Vγ2 and Vγ5 rearrange later in development (140).

E2A regulates ordered Vγ rearrangements (145, 146). In fetal thymocytes, both Vγ2 and Vγ3 genes have permissive chromatin states, and the rearrangement preference for Vγ3 depends on its more proximal location to Jγ1 (147–150). In adults thymocytes, selection of Vγ genes for rearrangement depends on the Vγ promoters (151). E2A regulates ordered rearrangement of Vγ genes by increasing chromatin accessibility at Vγ2 and reducing chromatin accessibility at Vγ3 in adult thymocytes. E2A and HEB bind directly to the Vγ2 gene *in vivo* and positively regulate GLTs from and histone acetylation at the Vγ2 gene in a dose dependent manner. E2A deficient mice have reduced Vγ2 rearrangements in both fetal and adult thymocytes. Further, E2A represses Vγ3 GLTs in adult mice, and E2A deficient mice have increased Vγ3 rearrangements in adult thymocytes. These results indicate that while E2A promotes Vγ2 rearrangement in both fetal in adult thymocytes, ordered rearrangement depends on specific repression of the fetal Vγ3 gene in adult thymocytes by E2A (145, 146).

### TCRδ Rearrangement

Unlike other antigen receptor loci composed of V, D and J gene segments, V_δ_ to D_δ_ rearrangement usually precedes D_δ_ to J_δ_ rearrangement (152). E2A has a role in promoting V_δ_-D_δ_ rearrangements, but not in D_δ_-J_δ_ rearrangements (145). Expression of E2A or HEB with Rag in non-lymphoid cells can induce V_δ_-D_δ_ rearrangements (144). Like in TCRγ development, the TCRδ locus rearranges particular V_δ_ genes at specific stages in development. Recombination of Vδ1 predominates in early fetal development, but Vδ1 rearrangement is rare in the adult thymus. Vδ5 rearrangement begins later in development and predominates in the adult thymus (152, 153). E2A acts to both positively and negatively regulate rearrangement of particular Vδ genes in adult and fetal thymocytes. E2A represses Vδ1 rearrangement in adult thymocytes in a dose dependent manner, and E2A deficient mice exhibit increased rearrangements involving Vδ1. E2A also promotes rearrangement of the predominantly adult gene Vδ5. Vδ5 rearrangements that usually predominate in the adult thymus are not present in E2A deficient mice. In E2A deficient mice, Vδ5 rearrangements are reduced fetal thymocytes in a dose dependent manner and in adult thymocytes in a dose independent manner (145).

## Modulation of Natural Killer T Cell Development and Rearrangement

While Id3 is generally involved in orchestrating γδ cell fate, the Id proteins restrict development of a specific subset of γδ T cells, γδ NKT cells. γδ NKT cells are innate-like γδ T cells that express a semi-invariant receptor (Vγ1.1Vδ6.3), and are associated with many innate like characteristics. Loss of Id3 expression in γδ T cells leads to higher E protein activity, upregulation of Egr2, PLZF, and c-Myc and proliferative expansion of γδ NKT cells (154). Id3 deficient mice also show an expanded population of γδ NKT cells (155–157). Id2 either cooperates with or can compensate for Id3, and γδ NKT cells are expanded even more so in Id3 deficient mice that also have compromised Id2 function (157). Deletion of Id2 promotes a smaller expansion of γδ T cells, although interestingly, this expansion of γδ is not limited to cells expressing Vγ1.1Vδ6.3 (157). Id2 and Id3 restrict development into the NKT γδ T cell fate by inhibiting E protein activity, and deletion of E proteins in Id deficient mice reverts the expansion of NKT γδ T cells (156, 157).

The mechanism by which inhibition of Id protein activity expands the γδ NKT population is unclear. It is possible that γδ NKT expansion could be a result of increased rearrangement, however there are conflicting findings regarding whether the expansion of Vγ1.1 γδ NKT cells in Id3 deficient mice occurs at the expense of other γδ T cells. It has been reported that Id3^-/-^ mice have reduced numbers of Vγ2 and Vγ3 dendritic epidermal T cell (DETC) subsets (116). Others note that the expanded use of the Vγ1.1 gene is not at the expense of other Vγ genes, and that the number of cells expressing Vγ2 and Vγ5 genes is the same, though the proportion of γδ T cells expressing them is reduced (158). One possible explanation for the expansion of γδ NKT cells is that these cells are normally deleted as a result of excessive γδTCR signaling, but Id3 deficiency allows them to escape deletion and proliferate (159).

**Figure 5:**
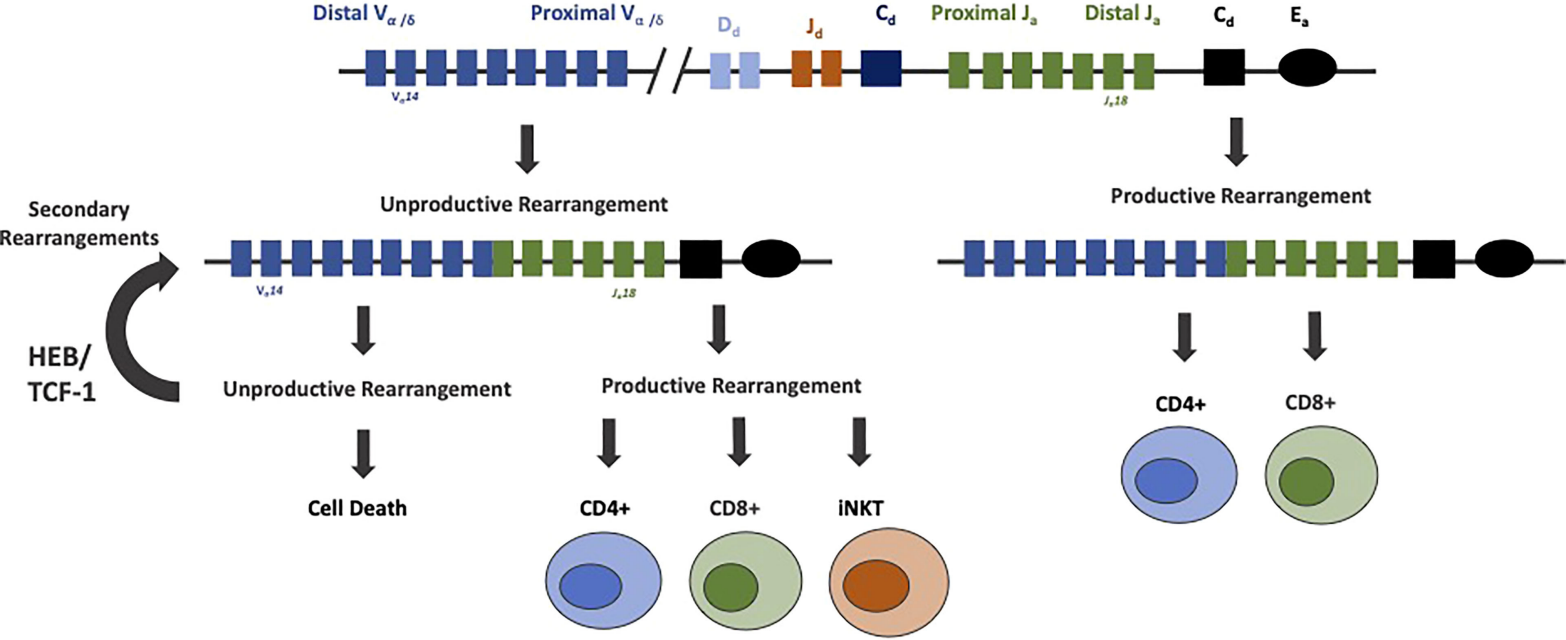
HEB prolongs survival of DP thymocytes and rearrangements of distal V_α_ and J_α_ gene segments. Rearrangement of the TCRα locus proceeds by a deletional mechanism, in which the more proximal gene segments rearrange first. Cells that undergo productive rearrangements that pass positive selection mature into CD4+ and CD8+ mature T cells. Cells with unproductive rearrangements undergo secondary rearrangements, until the cells either produce a productive TCRα allele or undergo cell death. HEB, along with TCF-1, promotes rearrangements of more distal TCRα genes by prolonging cell survival during this process. HEB is crucial for the rearrangement of distal TCRα genes, production of a diverse αβ T cell repertoire, and generation of iNKT cells expressing an invariant TCRα protein composed of the distally located V_α_14 and J_α_18 genes.

A small fraction of DP thymocytes differentiate into invariant NKT (iNKT) cells, driven by heightened E protein activity and modulation of Id2/3 protein expression (160). Upon positive selection, iNKT cells further mature into multiple subsets, including NKT1, NKT2 and NKT17 cells. These developmental transitions are again instructed by E-Id protein activity to indirectly impact CD8+ T cell fate (161–165). iNKT cells express an invariant TCRα chain composed of the distally located V_α_14-J_α_18 gene segments, which recombine in secondary TCRα rearrangements. Several rounds of Vα to Jα recombination occur during TCRα rearrangement. Primary rearrangements of the TCRα locus make use of the most proximal 3’ Vα genes and most distal 5’ Jα genes. Secondary rearrangements make use of more 5’ Vα and 3’ Jα segments. Recombination is terminated when cells either pass positive selection or undergo cell death. Prolonged survival at the DP stage allows cells to undergo more sequential arrangements before undergoing cell death. HEB cooperates with TCF-1 to promote the survival of DP thymocytes by positively regulating the anti-apoptotic gene Bcl-XL (118, 166–169). DP thymocytes which lack HEB have an impaired ability to survive, rearrange their distal Jα genes less, and completely lack iNKT cells. This loss of iNKT cells is attributed to the shortened lifespan of DP cells and subsequent deficiencies in secondary TCRα rearrangements, as ectopic expression of Bcl-XL restores secondary TCRα rearrangements and iNKT development (118). Taken together, these data indicated that HEB instructs the generation of a diverse αβ T cell repertoire, enabling usage of all distally located genes and the development of iNKT cells (**Figure 5**).

## Modulation of Mature T Cell Development

E protein activity mediates the development of DP thymocytes into unique developmental fates within the CD4+ or CD8+ T cell compartments. The role of E2A and HEB in supporting the development of an appropriate ratio of CD4+ to CD8+ T cells is now well established (170–172). These E proteins bind to CD4 E-box site to support CD4+ development while antagonizing Id2/3 activity is required for CD8+ development (171–173). E proteins mediate this development by modulating CCR7 and IL7Rα expression (171). Conversely, this activity is suppressed by Id proteins to guide CD8+ development (171). After the successful rearrangement of the TCRβ and TCRα loci, TCR-signaling induces Id3 expression, which is then maintained to enforce a naïve state in peripheral T cells (174). Id2 is then upregulated at a later stage through an unknown pathway. Id2 was found to downregulate Id3 while Id3 had no effect on Id2 expression, indicating that some of the Id2-mediated effects on gene expression may be indirect (175). In summary, sustained sequestration of E proteins by Id proteins may maintain thymic single positive T cells in a naïve state.

CD4+ T cells are instructed towards unique developmental fates by the delicate balancing and timing of E protein activity. Unopposed E protein activity readily leads to the development of innate variant follicular helper T cell (T_FH_) cells (174). In peripheral CD4+ T cells, Id2 and Id3 act to support the Th1 development while restraining T_FH_ lineage differentiation (176). In parallel studies it was shown that Id2 suppresses T_FH_ development and expansion by activating the PI3K–AKT–mTORC1–Hif1a and c-myc/p19Arf pathways (173, 177). An alternative pathway that underpins T_FH_ cell development may involve the induction of Bcl6 expression, which in turn inhibits Id2 expression (176). By permitting T_FH_ development, E proteins also influence the formation of germinal centers, with higher amounts of GC and PC B cells found in both thymi and peripheral lymphoid organs derived from mice that harbor Id2/3 deletions (173, 174). These findings highlight the importance of E protein activity in T cells in coordinating a B cell response and for germinal center adaptive immune cell development. Id proteins also orchestrate developmental progression of Treg cells (178, 179). Upon depletion of Id2 and Id3 expression in Treg cells mice readily develop Th2-cell mediated inflammatory disease (178–180). Collectively these studies revealed that E and Id proteins modulate the development of an ensemble of distinct peripheral CD4+ T cells to combat infection and suppress the development of autoimmune disease.

Id2 and Id3 also regulate E protein activity to instruct CD8+ T cell development. Naïve CD8+ T cells stimulated by the appropriate antigen readily elevate E-protein DNA binding (181). A series of elegant studies revealed that the E-Id protein axis also controls the developmental progression of CD8+ effector and memory T cells (175, 182–186). High levels of Id2 expression are required to instruct CD8+ effector T differentiation while suppressing the development of CD8+ memory cells (182, 183, 185). Conversely, upregulated Id3 expression promoted the development of Cd8+ memory cells (175). Id proteins further perform a key role in orchestrating the development of long-lived resident memory (Trm) cells (187). In summary, E and Id proteins play critical roles in orchestrating the development of an ensemble of immune cell types that act collectively to combat infection.

## Transcriptional Bursting and RNA Decay Pathways Dictate E2A and E2-2 mRNA Heterogeneity

Very early studies revealed that E47 protein abundance is noisy in naïve B cells. While a small proportion of naïve B cells express detectable levels of E47, E47 abundance is uniformly high in activated B cells (90). Consistent with these observations, more recent studies showed that E47 mRNA abundance varied across the naïve B cell population while heterogeneity in E47 mRNA levels in activated B cells was low (188). These findings raised the question as to how such differences in mRNA abundance and heterogeneity are established. Quantitative studies have addressed this question (188). E2A and E2-2 bursting frequencies and mRNA life-times differ between naïve and activated B cells. In naïve B cells, E2A and E2-2 bursting frequencies are low and mRNA life-times are short. Conversely in activated B cells E2A and E2-2 bursting frequencies are high and mRNA life-times are long (188). These findings bring into question how alterations in E2A and E2-2 mRNA life-times are established. One possible mechanism involves miRNA instructed fine-tuning of E2A and E2-2 mRNA abundance, and it will be important to identify potential miRNAs that target HLH genes. Finally, we would like to consider a role for heterogeneity in E2A and E2-2 mRNA abundance in instructing lymphocyte activation. We suggest that upon interacting of the BCR with invading pathogens, E2A and E2-2 heterogeneity in mRNA abundance permits a swift and clonal response. In such a scenario, the few B naïve cells that are actively bursting across the B cell population are primed to readily undergo CSR or rapidly develop into differentiating plasma cells. Conversely, increased E2A and E2-2 bursting frequencies and lower mRNA decay rates in activated B cells may decrease heterogeneity in E2A and E2-2 abundance to orchestrate B cell maturation. We propose that similar mechanisms instruct the immune response in T cells. Upon viral or tumor encounters the decision to differentiate into effector or memory T cell fate is similarly dictated by the combined alterations in E2A, E2-2 and HEB bursting frequencies and mRNA life-times.

## Conclusion

Over three decades of research have highlighted critical functions of E- and Id-proteins in instructing adaptive immune development. E-proteins activate B- and T-lineage specific gene programs to specify B and T cell fate. They promote the assembly of antigen receptor loci to generate a diverse antibody and TCR repertoire. In maturing thymocytes, the E- and Id-proteins promote thymocyte selection. In peripheral B and T cells, the rise and fall in E- and Id-proteins orchestrate the development of an array of regulatory, effector and memory cell types. In mechanistic terms, E-proteins sequester histone acetyltransferases across the enhancer landscape to promote the deposition of H3K27Ac. The deposition of H3K27Ac, in turn, initiates loop extrusion to assemble a wide ensemble of loops across antigen receptor loci and down-stream target genes. We suggest that these proteins also assemble loop domains into nuclear condensates to regulate antigen receptor loci rearrangement and lineage specific programs of gene expression. Finally, we propose that alterations in HLH bursting frequencies and mRNA life-times increase and/or narrow heterogeneity in mRNA abundance to establish B or T cell identity, thereby instructing the developmental progression of peripheral effector and memory lymphocytes in response to invading pathogens.

## Author Contributions

MA and ZW wrote the manuscript and designed figures with inputs from CM. All authors contributed to the article and approved the submitted version.

## Funding

ZW was supported by a David V. Goeddel Endowed Graduate Fellowship and by a training grant from the NIH (2T32DK007541-31A1). CM was supported by the NIH AI00880, AI09599, AI102853 and AI102853.

## Conflict of Interest

The authors declare that the research was conducted in the absence of any commercial or financial relationships that could be construed as a potential conflict of interest.

## Publisher’s Note

All claims expressed in this article are solely those of the authors and do not necessarily represent those of their affiliated organizations, or those of the publisher, the editors and the reviewers. Any product that may be evaluated in this article, or claim that may be made by its manufacturer, is not guaranteed or endorsed by the publisher.
